# Recreational activity after open hip abductor repair

**DOI:** 10.1007/s00402-022-04734-5

**Published:** 2022-12-22

**Authors:** Luis Navas, Alexander Zimmerer, Matthias Hausschild

**Affiliations:** 1 Department of Orthopaedic and Trauma Surgery, Orthopädische Klinik Paulinenhilfe, Diakonieklinikum, Rosenbergstrasse 38, 70192 Stuttgart, Germany; 2 ARCUS Sportklinik, Rastatterstraße 17-19, 72175 Pforzheim, Germany; 3 Department of Orthopaedics and Orthopaedic Surgery, University Medicine Greifswald, Ferdinand-Sauerbruch-Straße, 17475 Greifswald, Germany

**Keywords:** Gluteal muscles, Return to activity, Hip abductor tear, PROM

## Abstract

**Background:**

Hip abductor tear (HAT) is an increasingly diagnosed cause of refractory lateral hip pain and dysfunction, affecting 10–25% of the general population.

**Purpose:**

(1) to determine the rate of return to activity and to assess the physical and recreational activity of patients undergoing open hip abductor repair (oHATr) and (2) to describe the modification or initiation of new sports disciplines.

**Study design:**

Case series; Level of evidence, 4.

**Methods:**

A total of 28 patients (29 hips) who underwent an oHATr were prospectively analyzed at a midterm follow-up of 3.5 (range 2–5) years. The sports and recreational activity levels, as well as type of sports practiced before and after surgery, and The Veterans RAND 12 Item Health Survey (VR-12) were assessed via questionnaire.

**Results:**

At the final follow-up, all patients were active in sports after surgery. The duration and frequency of sports activities showed a slight decrease (48–42 min per week and 3.2–2.9 sessions per week, respectively) (*p* = 0.412 and 0.135, respectively). The VR-12 had a score of 45 (13.12–63.18) points for the physical component and 41 (32–53.8) points for the mental component. 100% of the patients would undergo the surgery again. 95% of patients were satisfied with the overall results of the surgical outcome, with 98% satisfied with their hip pain relief and ability to undertake daily and work activities. Moreover, 94% were satisfied with their ability to return to recreational activities. The failure rate in our cohort was approximately 14%.

**Conclusion:**

All patients who underwent an oHATr were able to return at least to one type of sport. This cohort was highly satisfied with their sports involvement and recreational activity achievement. In addition, 88% of patients reported that oHATr improved sports activity. There was a shift from higher to lower impact sports. Furthermore, just 3 hips present a retear after surgery.

## Background

Hip abductor tear (HAT) is an increasingly diagnosed cause of refractory lateral hip pain and dysfunction [1–6], affecting 10–25% of the general population [2, 7–12].

HAT is usually caused by degeneration, direct trauma, iatrogenic injury during hip surgery (e.g., total hip arthroplasty (THA) using direct lateral approaches), and tissue damage by the presence of metal ions from metal-on-metal THA [13–16]. The patients with HAT present with lateral hip pain, tenderness to palpation of the greater trochanter, weakened hip abduction on strength testing, and a positive Trendelenburg-Sign on gait examination. These symptoms are aggravated by long walks, climbing and descending stairs, and sleeping on the affected side [17–19].

Systematic reviews report a higher number of surgical complications associated with open repair techniques, although there is no difference in strength development or clinical scores [8, 17, 20].

A review of surgical repair methods reported that many of these studies reporting outcomes after HAT repair were missing some detail on the patient cohort, surgical technique, post-operative care, and clinical follow-up [21–23].

Therefore, the aim of this study was (1) to determine the rate of return to activity and to assess the physical and recreational activity of patients undergoing open hip abductor repair and (2) to describe the modification or initiation of new sports disciplines. We hypothesized that patients treated with an open hip abductor tear repair would be able to return to usual recreational activity.

## Materials and methods

### Patient selection

The present single-center retrospective study comprises a cohort of 28 consecutive patients with a mean age of 60 (29–85) years following an open HAT repair (oHATr) performed by the senior author (M.H.) between March 2016 and March 2020. We identified patients through our institutional database and performed a retrospective analysis of prospectively collected data via questionnaire.

**Fig. 1:**
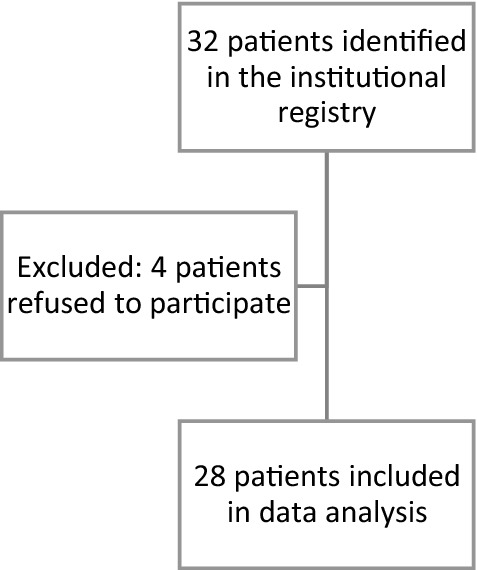
Patient inclusion/exclusion flowchart

Indications for oHATr included lateral hip pain, weakened abduction on physical examination, Trendelenburg gate, magnetic resonance imaging (MRI) findings consistent with full-thickness gluteus medius and/or minimus tear in the posterior and lateral facet, and failure of at least 6 months of non-operative therapy, including non-steroidal anti-inflammatory drugs (NSAIDs), platelet-rich plasma (PRP) infiltration, and physical therapy. Exclusion criteria included a history of pediatric hip malformations, prior surgery of ipsilateral HAT, partial-thickness gluteus medius and/or minimus tears, a follow-up period shorter than 12 months, or inability to consent to the study. Complication data were collected by reviewing the electronic medical records at our Centre. The clinical examination was performed by two fellowship-trained orthopedists (M.H., A.Z.). Likewise, MR imaging was assessed by both examiners. The data were analyzed by calculating intraclass correlation coefficients (ICCs). We found excellent inter-observer agreement in classifying the tear types (ICC, 0.98). The patient enrollment flowchart is demonstrated in Fig. 1.

Patients were asked for their consent to participate in the study and for their pre- and post-operative data to be prospectively recorded in a secure institutional repository.

The ethics commission (Ethikkommission der Landesärztekammer Baden-Württemberg Germany, F-2019-006) approved all procedures, and the study was conducted in accordance with the Helsinki Declaration of 1975, as revised in 2008. All patients gave informed consent.

### Surgical technique

The surgical refixation of the gluteal tendon tears was performed by mini-open double-row technique and conducted by the senior author (M.H.) [24]. The surgery was executed in a lateral decubitus position under general anesthesia. A 6–8 cm lateral approach was used directly over the greater trochanter and iliotibial band (ITB). After a longitudinal incision through the ITB, the peritrochanteric space was reached, the trochanteric subgluteal bursa was removed and the tear was identified, then a longitudinal incision of the gluteal tendons was performed over the tear.

This was followed by debridement and mobilization of the tendons to achieve an adequate distalisation to the tendon footprint on the posterior and lateral facet of the trochanter, debridement of the sclerosis on the greater trochanter, drilling and tapping of the proximal anchor row. Two 3.5 mm SwiveLock^®^ anchors (Arthrex, FL, USA) loaded with nonabsorbable suture strips were then proximally placed. The sutures were then passed through the tendon in a fan-shaped pattern. Once passed, the suture strips are crossed in a double V-shape and locked with 4.75 mm SwiveLock^®^ anchors in the distal row under slight pre-tensioning of the gluteal tendons.

The ITB was closed with 2-vicryl sutures. The subcutaneous tissue was closed with 2–0 vicryl sutures, and the skin was sutured with a continuous subcuticular 3–0 monocryl suture. The hip was softly adducted and abducted using a brace to ensure adequate tension of the repair.

### Post-operative management

The postoperative rehabilitation program was standardized for all patients. Patients received a hip brace for the first six weeks postoperatively to restrict external rotation and abduction. Partial weight-bearing was limited to 20 kg. Patients were able to bear the full weight for the next six weeks and began hip stabilization and strengthening exercises while the brace was removed. After 12 weeks, patients were permitted to walk freely and return to activities they tolerated in a pain-adapted form. Deep vein thrombosis prophylaxis was indicated until full weight bearing was achieved.

### Patient-reported clinical outcomes

The sports and recreational activity levels, as well as type of sports practiced was recorded before the occurrence of the first symptoms and at the follow-up time.

The Veterans RAND 12 Item Health Survey (VR-12) evaluated the general health of the patient producing a mental (MCS) and physical component subscale (PCS).

### Statistical analysis

Means and standard deviations have been reported for continuous variables. According to the Shapiro–Wilk test, the study cohort was normally distributed (*p* = 0.257). Differences between pre-and postoperative data were examined with a paired *t*-test and Wilcoxon signed-rank test. McNemar’s test statistic was conducted to detect differences. Statistical analyses were conducted using SPSS statistical software (IBM SPSS Statistics for Windows, version 26.0.0; IBM Corp).

## Results

### Demographics

Twenty-eight patients (29 hips) were included in the analysis. The mean age was 59.8 ± 12.5 (29–85) years and the mean Body Mass Index (BMI) was 28 ± 4.5 (20.2–35.3) kg/m^2^. Surgery was performed on 24 women and 4 men (Table 1). The mean follow-up was 40.5 ± 26.6 (22–67) months.

**Table 1:** Patient demographic data

	Value
Total no. of patients	28 (29 Hips)
Laterality, *n* (%)	
Right	15 (52%)
Left	14 (48%)
Gender, *n* (%)	
Female	24 (85.7%)
Male	4 (14.3%)
Age, y	59.79 ± 12.45 (29–85)
Body mass index, kg/m^2^	27.99 ± 4.45 (20–35)

Values are shown as *n* (%) or mean ± SD (range)

Re-tear occurred in three hips during the follow-up, and one surgical site infection was observed in one patient, which required surgical intervention.

### Sports and recreational activity

Complete information from the questionnaire was available for all 28 patients. After surgery, all patients (100%) were active in at least one sporting activity. Patients practiced an average of three sports at the last follow-up, which was significantly different from the number of sports they practiced before the onset of the first symptoms (5.5 sports; *p* < 0.0001). A significant decrease in biking, hiking, alpine skiing and jogging after surgery could be shown (Table 2). As a reason for less physical activity, 55% stated that they were afraid of re-injury, 16% were more anxious, 15% on the advice of their physiotherapist and 14% were less physically competent.

**Table 2:** Sport disciplines before and after oHATr

Discipline	Prior to oAHTr (%)	After oHATr (%)	*p* value
Biking	20 (18.4%)	13 (20.6%)	**0.016**
Hiking	15 (13.8%)	6 (9.5%)	**0.013**
Alpine skiing	6 (5.5%)	0	**0.031**
Jogging	8 (7.3%)	1 (1.6%)	**0.016**
Soccer	4 (3.7%)	0	0.133
Tennis	4 (3.7%)	3 (4.8%)	1
Nordic-Walking	10 (9.2%)	9 (14.2%)	1
Hand-, Volley-, Basketball	1 (1%)	0	1
Long walks	20 (18.3%)	15 (23.8%)	0.074
Cross-country skiing	3 (2.8%)	0	0.248
Fitness training	10 (9.1%)	10 (15.9%)	1
Swimming	5 (4.4%)	6 (9.5%)	1
Horse riding	3 (2.8%)	0	0.248

Values are shown as *n* (%)

Significant *p* values are in bold

### Frequency and intensity of sport sessions

**Fig. 2:**
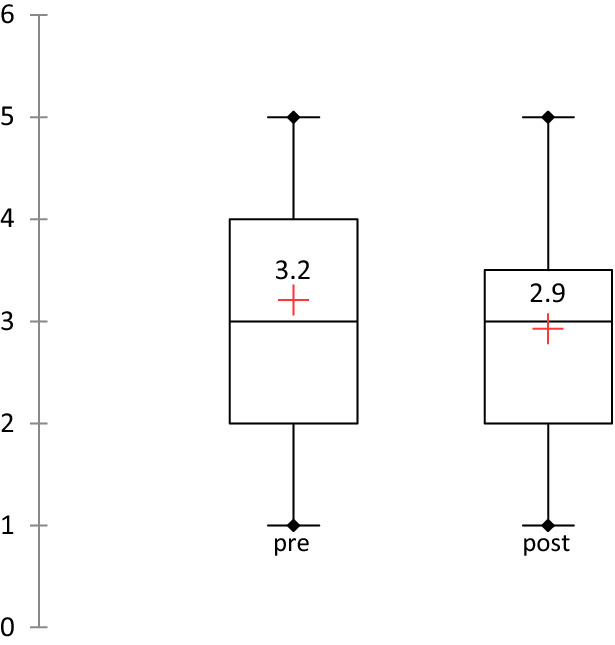
Number of sport sessions per week before and after oHATr. The values are shown as the mean values (*p* = 0.412)

The frequency (sport sessions per week) did not increase from the level before surgery (*p* = 0.412) (Fig. 2).

**Fig. 3:**
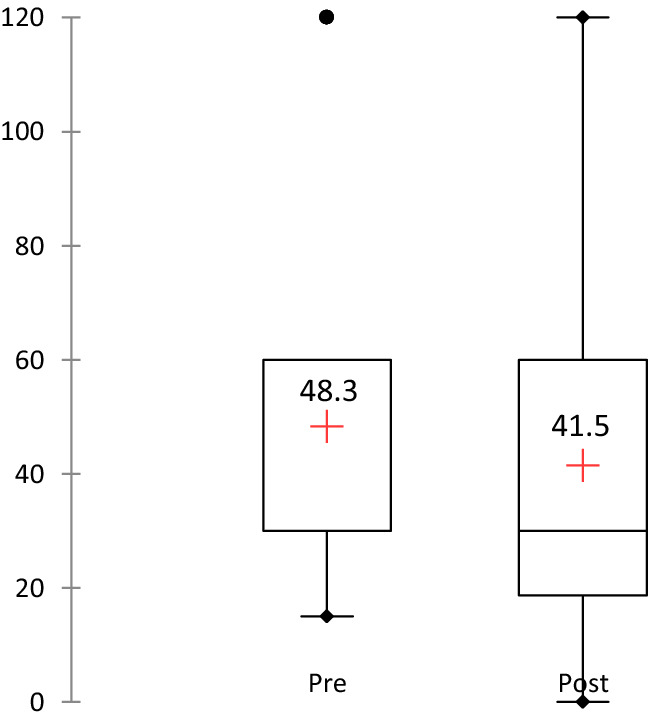
Session length per week before and after oHATr. The values are shown as the mean values (*p* = 0.135)

The minimum session length per week decreased from 48.3 ± 29.5 (15–120) min before surgery to 41.5 ± 33.2 (0–120) min at the last follow-up (*p* = 0.135) (Fig. 3).

Sixty percent of patients returned to sports activities within 1 month after surgery, 26% resumed sports activities within 3 months, and 14% resumed their physical activities after 6 months (surgical reintervention group).

### Outcome scores

Paired *t*-test analysis of preoperative and postoperative reported outcomes demonstrated statistically significant improvements in UCLA (3.8 ± 1.7 vs 5 ± 1.5; *p* < 0.0001) score. Postoperatively, only 4 patients (14%) reported a UCLA score ≥ 7, corresponding to highly active in sport activities. The Veterans RAND 12-Item Health Survey (VR-12) had a score of 45 ± 15 (13.12–63.18) points for the physical component and 41 ± 5.1 (32–53.8) points for the mental component (Table 3).

**Table 3:** Pre- and postoperative patient-reported outcomes

Score	Preoperative	Postoperative	*p* value
UCLA	3.8 ± 1.7 (1–9)	5 ± 1.5 (2–8)	< 0.0001
VR-12 (M)	21.15 ± 12 (8.4–43.19)	45 ± 15 (13.12–63.18)	< 0.0001
VR-12 (P)	35.5 ± 8 (23.1–45.2)	41 ± 5.1 (32–53.8)	< 0.0001

Values are shown as *n* (%) or mean ± SD (range). UCLA, the University of California and Los Angeles activity scale; VR-12, Veterans RAND 12-Item Health Survey

100% of the patients would undergo the surgery again. 95% of patients were satisfied with the overall results of the surgical outcome, with 98% satisfied with their hip pain relief and ability to undertake daily and work activities. Moreover 94% were satisfied with their ability to return to recreational activities.

## Discussion

In this study, we were able to demonstrate that patients who underwent open HAT repair were very satisfied with their sport and recreational activities. Hundred percent of the patients were active again in at least one recreational activity after oHATr.

In general, women are more likely to suffer hip abductor tears than men. A possible explanatory approach could be related to (1) anatomy, as women have a widened pelvic rim that alters ITB traction, (2) physiology, where hormonal effects may cause bursal irritation or pain generators, and/or (3) activity differences between men and women [5]. Although several surgical procedures have been published for the treatment of these tears [22, 25], studies often lack detailed information on patient population, postoperative care and clinical follow-up. To date, three studies have published results in more than 25 patients [21, 23, 26].Two systematic reviews comparing open and endoscopic repair found that both techniques produced similar improvements in PROs, pain scores, and abduction strength, with open repairs having a higher complication rate, including increased retear rate [8, 17]. In our cohort, three hips (10%) presented a retear after surgery.

In most cases, patients who decided to undergo a surgical procedure usually expected to improve their activity level. The development of surgical techniques and implants has led to an improvement of the patient-reported outcome measures (PROMs), with substantial attention from orthopedic surgeons given to returning to or starting new sport disciplines by patients.

In our cohort, a rate of return to sport activities of 100% was found at the last follow-up. In addition, 88% of patients reported that oHATr improved sports activity. These data are in superior way to the numbers published in a comparative study between open and endoscopic HAT repair [27]. This may be attributable to the fact that our cohort is younger than the one mentioned in the previous study.

In our cohort, preoperatively, only a few patients were practicing high-impact sports such as jogging, volleyball, and skiing, presumably because of the presence of symptoms of HAT. Interest in and participation in low-impact sports such as hiking, fitness training, and biking increased after surgery, in accordance with previous studies [27]. However, postoperatively, an increase in both high- and low-impact sports was not observed, while no reports of high-impact sports are available in the literature [28–31]. By the fact of not having a guideline which types of sport are considered high- or low-impact, we rely on the AHKS guidelines for total hip arthroplasty, the practice of low-impact sports is recommended without any previous sports experience or supervision, while medium- or high-impact sports are recommended only with previous sports experience or supervision. In accordance with the AHKS guidelines, most of our patients practiced low-, medium- or high-impact sports. However, just 2% of our patients practiced sports that were classified as not recommended even with or without previous experience or supervision (jogging). Based on our own experience, we do not recommend or prohibit specific sports disciplines. Nonetheless, patients are informed of general and sport-specific risks of higher activity and impact levels, such as possible increased retear risk. We observed no conversion of low-impact to intermediate/high-impact sports practices after surgery.

Overall, the significant postoperative clinical improvement correlated with the high level of satisfaction reported by patients in this study. At the last follow-up 95% of patients were satisfied with the overall results of the surgical outcome, with 98% satisfied with their hip pain relief and ability to undertake daily and work activities. Moreover 94% were satisfied with their ability to return to recreational activities.

A significant improvement in the severity of pain was stated in this study. The VAS for pain has been reported in several studies reporting the outcomes of HAT repair [21, 32–36] and our outcomes are also consistent with those previously described.

### Limitations

First, selection bias is possible because of the retrospective nature of this study. Second, there is currently only one preliminary study in the literature reporting these clinically meaningful outcome scores, type of sports practiced, as well as sport and recreational activity levels. Third, there was not a quantitative measurement of hip abduction strength before and after surgical repair. Fourth, the outcomes from this study were derived from self-reported data. However, patient-reported data are among the most important outcomes in orthopedic patients, and data collection was limited by the patient’s veracity, subjective opinion, and ability to remember information from several years prior.

## Conclusions

All patients who underwent an oHATr were able to return at least to one type of sport. This cohort was highly satisfied with their sports involvement and recreational activity achievement. In addition, 88% of patients reported that oHATr improved sports activity. Moreover, there was a shift from higher impact sports to lower impact sports.
